# Single column locking plate fixation is inadequate in two column acetabular fractures. A biomechanical analysis

**DOI:** 10.1186/1749-799X-5-30

**Published:** 2010-05-09

**Authors:** Kiarash Khajavi, Arthur T Lee, Derek P Lindsey, Philipp Leucht, Michael J Bellino, Nicholas J Giori

**Affiliations:** 1 Department of Orthopaedic Surgery, Stanford University School of Medicine, 300 Pasteur Drive, Stanford, CA 94305, USA; 2 Bone and Joint Center of Excellence, VA Palo Alto Healthcare System, 3801 Miranda Ave., Palo Alto, CA 94304, USA

## Abstract

**Background:**

The objective of this study was to determine whether one can achieve stable fixation of a two column (transverse) acetabular fracture by only fixing a single column with a locking plate and unicortical locking screws. We hypothesized that a locking plate applied to the anterior column of a transverse acetabular fracture would create a construct that is more rigid than a non-locking plate, and that this construct would be biomechanically comparable to two column fixation.

**Methods:**

Using urethane foam models of the pelvis, we simulated transverse acetabular fractures and stabilized them with 1) an anterior column plate with bicortical screws, 2) an anterior locking plate with unicortical screws, 3) an anterior plate and posterior column lag screw, and 4) a posterior plate with an anterior column lag screw. These constructs were mechanically loaded on a servohydraulic material testing machine. Construct stiffness and fracture displacement were measured.

**Result and Discussion:**

We found that two column fixation is 54% stiffer than a single column fixation with a conventional plate with bicortical screws. There was no significant difference between fixation with an anterior column locking plate with unicortical screws and an anterior plate with posterior column lag screw. We detected a non-significant trend towards more stiffness for the anterior locking plate compared to the anterior non-locking plate.

**Conclusion:**

In conclusion, a locking plate construct of the anterior column provides less stability than a traditional both column construct with posterior plate and anterior column lag screw. However, the locking construct offers greater strength than a non-locking, bicortical construct, which in addition often requires extensive contouring and its application is oftentimes accompanied by the risk of neurovascular damage.

## Introduction

Intraarticular acetabular fractures are commonly treated with open reduction and internal fixation. Transverse acetabular fractures, as defined by Letournel and Judet [1], extend intraarticularly across both the anterior and posterior column of the pelvis, and divide the pelvis into a superior segment containing the roof and intact ilium and an inferior segment consisting of a single ischio-pubic segment. Internal fixation of these fractures often involves a combination of plates and screws to maintain perfect reduction. Fixation may involve plating of the anterior column and posterior column, or plating of one column in conjunction with lag screw fixation of the opposite column. In a biomechanical analysis, Shazar et al. showed that plating of one column in conjunction with lag screw fixation of the opposite column provided the stiffest construct compared to plating of a single column [2].

Locking plates have recently been developed for the internal fixation of fractures, and are gaining widespread acceptance. Locking plates have several advantages over traditional screw/plate constructs. There is improved angular stability because each screw acts as a small fixed-angled device. One can thus obtain better fixation in osteoporotic bone, and there is the opportunity to use unicortical, rather than bicortical screws [3-5]. Because fixation does not depend on friction between the plate and bone, one can apply plates with less disruption to periosteal blood supply and potentially improve the biological environment for fracture healing [3,6-8].

There are no current studies that have investigated the application of locking plates to acetabular fractures, and in particular, transverse acetabular fractures. We set out to compare the biomechanical stability of locking pelvic reconstruction plates with constructs that have previously been tested in the literature (i.e. non-locking plates as well as constructs that provide fixation of both the anterior and posterior columns). We hypothesize that a locking plate applied to the anterior column of a transverse acetabular fracture will result in a construct that is more rigid than a non-locking plate, and that this construct would be biomechanically comparable in stiffness and stability to two column fixation.

## Materials and methods

**Figure 1:**
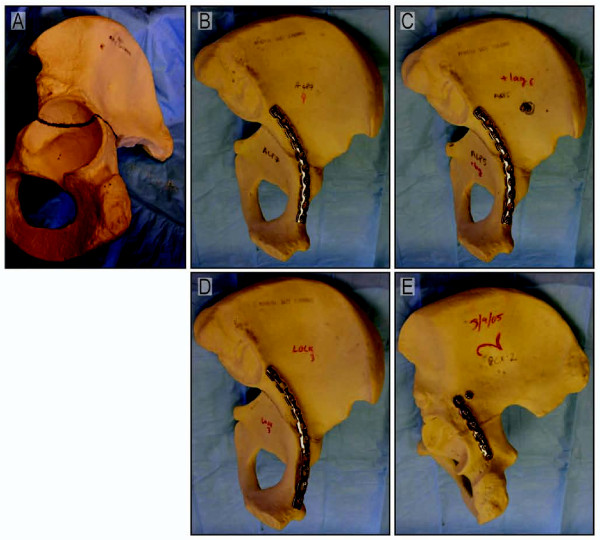
**(A) A urethane foam pelvis used in this study is shown with a line demonstrating the location of the simulated transverse acetabular fracture**. (B) A 10 hole 3.5 mm anterior column reconstruction plate with three bicortical screws on either side of the osteotomy (ACP). (C) A 10 hole 3.5 mm anterior column reconstruction plate with three bicortical screws on either side of the osteotomy and a 4.5 mm/120 mm posterior column lag screw (ACPLS). (D) A 10 hole 3.5 mm anterior column locking reconstruction plate with three unicortical screws on either side of the osteotomy (LOCK). (E) A 6 hole 3.5 mm posterior column reconstruction plate with three bicortical screws on either side of the osteotomy and a 4.5 mm/120 mm anterior column lag screw (PCPLS).

Forty urethane foam hemi-pelvises (Pacific Research Laboratories, Vashon, Washington), each with a well defined cortical outer shell and cancellous inner matrix were randomly divided into four groups of ten. Urethane foam hemi pelvises were chosen to control for the variability in cadaveric specimens as well as for the large number of specimens needed based on our power analysis. An identical transtectal osteotomy using a hand held saw was performed on each of the specimens (Fig. 1a). The osteotomy began at the mid portion of the greater sciatic notch and traveled across the posterior column, through the roof of the acetabulum, exiting through the anterior column at the level of the iliopectineal eminence.

The osteotomy was reduced anatomically and fixed in one of four ways: 1) a 10 hole 3.5 mm anterior column reconstruction plate with three bicortical screws on either side of the osteotomy (ACP), 2) a 10 hole 3.5 mm anterior column locking reconstruction plate with three unicortical screws on either side of the osteotomy (LOCK), 3) a 10 hole 3.5 mm anterior column reconstruction plate with three bicortical screws on either side of the osteotomy and a 4.5 mm/120 mm posterior column lag screw (ACPLS), and finally 4) a 6 hole 3.5 mm posterior column reconstruction plate with three bicortical screws on either side of the osteotomy and a 4.5 mm/120 mm anterior column lag screw (PCPLS) (Fig 1b-e).

**Figure 2:**
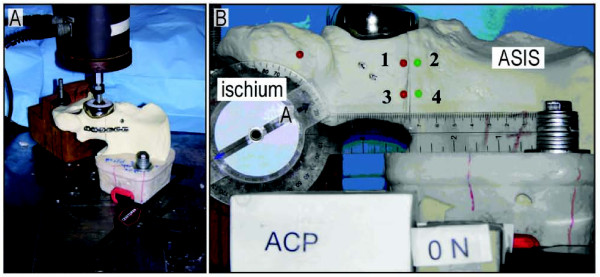
**(A) The testing apparatus consists of a bipolar hemiarthroplasty attached to a servohydraulic materials testing machine (858 Mini Bionix, MTS, Eden Prairie, MN)**. The customized jig was oriented to allow femoral head loading to be oriented 45 degrees superomedially (coronal plane) and 25 degrees posteriorly. (B) The hemiarthroplasty head is in the acetabulum at the top of the figure. To the left is the ischium and to the right is the ilium. The numbered pins were used to record motion at the fracture site.

Each specimen was stabilized in a customized jig (Fig 2a). A PMMA mold stabilized the superior osteotomy fragment (i.e. the intact ilium) and was bolted to the testing table for stability. To enforce an anatomic boundary condition at the pubis, the pubic symphysis rested on a block of wood that was cut at an angle that matched the anatomical mid-sagittal plane. Thus, the only constraint to motion of the inferior portion of the pelvis was that the pubic symphysis portion of the hemipelvis could not cross the mid-sagittal plane of the body. It was otherwise free to translate and rotate in all other directions.

A bipolar hemiarthroplasty was attached to a servohydraulic material testing machine (858 Mini Bionix, MTS, Eden Prairie, MN) in order to load the construct. The customized jig was oriented to allow femoral head loading to be oriented 45 degrees superomedially (coronal plane) and 25 degrees posteriorly (sagittal plane) [1,9,10].

Prior to specimen loading four markers were attached to each osteotomized urethane foam pelvis to allow measurement of the relative motion across the osteotomy (Fig. 2b). These markers were placed along the posterior column of the pelvis adjacent to the osteotomy gap. The markers were placed 5 mm from the gap on each side of the fracture line and were placed 2 cm apart. Two opposing markers (numbers 1 and 2) were in a more anterior position along the osteotomy line, while the other two opposing markers (numbers 3 and 4) were in a more posterior position. A photograph was taken with a digital camera (Coolpix 8700; Nikon) attached to a tripod in the unloaded state with a ruler in the field of view to allow for subsequent calibration. Specimens were then loaded at 0.2 mm/sec to 1000N and another photograph was taken. Lastly, the specimens were loaded up to 2000N while piston displacement and load were acquired and then a final photograph was taken. To avoid the effects of the toe region, stiffness of the construct was calculated between 1000 and 2000 N, where the load-displacement curve was most linear.

Marker positions from the three images were analyzed using ImageJ (http://rsb.info.nih.gov/ij/; NIH, Bethesda, MD). The four markers were used to define how the gap opened at 2000N relative to 0N for the four plated constructs. Displacements at two points along the fracture line were defined. The anterior displacement was defined as the movement of pin 1 relative to pin 2, and the posterior displacement was defined as the movement of pin 3 relative to pin 4.

To represent overall motion of the fracture fragments at the fracture site, the average location of each pin in space for each fixation scheme was graphed. Visualizing the displacements in this way allows one to understand how the fracture displaced under load, either perpendicular to the fracture line and creating a gap, or parallel to the fracture line and generating shear.

Differences in stiffness of the various plating constructs were then analyzed using an ANOVA test. Assuming a stiffness standard deviation of 0.25 N/mm and a difference desired to detect of 0.5 N/mm, we calculated that 10 specimens per group would give a power of 0.9986. Standard deviation and mean values were based on a previous study [10].

## Results

### Analysis of construct stiffness

**Figure 3:**
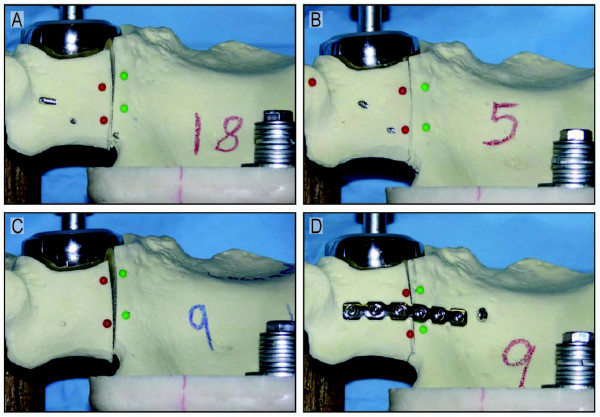
**Representative photos of displacement at the fracture site with all four fixation schemes at 2000 N of loading are shown**. (A) Anterior column plate; (B) Anterior column plate with posterior column lag screw; (C) Anterior column locking plate; (D) Posterior column plate with anterior column lag screw.

**Figure 4:**
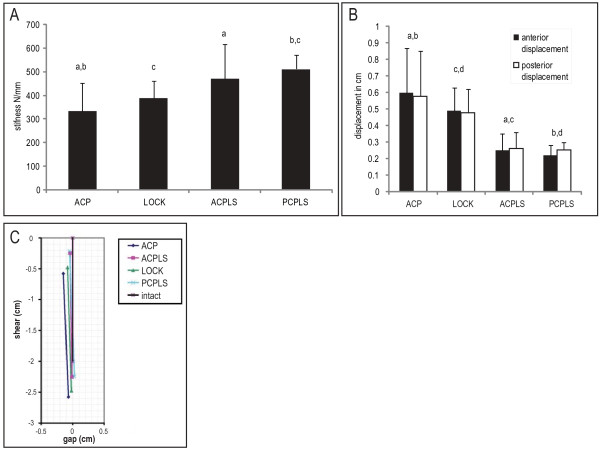
**(A) Mean stiffness (N/mm) and standard deviations for different fixation modalities**. (B) Displacements measured for the anterior (black) and posterior (white) gaps during loading from 0 to 2000 N for four different pelvic fracture fixation modalities. Values with common superscripts are significantly different (p < 0.05). (C) A diagram representing average displacement at the fracture site for the four different fixation schemes is shown. The reference superior edge of the fracture line is represented by the solid vertical black line in the central-upper part of this figure. The colored lines to the left and below this reference line represent the average location of the opposing inferior fracture edges after load is applied, and are based on the movement of the pins in the photographs as seen in Figure 3. Motion in the -X direction represents opening of the fracture gap and -Y represents shear motion at the fracture site. The single column fixation schemes (ACP and LOCK) displaced approximately twice as much as the two-column fixation schemes (ACPLS and PCPLS).

In order to test our hypothesis that a locking plate applied to the anterior column of a transverse acetabular fracture will result in a construct that is more rigid than a non-locking plate and that this construct would be biomechanically comparable in stiffness to two column fixation, we loaded the four fixation construct with the above described protocol. Typical photos of the fracture site at 2000 N loading with the four fixation schemes are shown in Figure 3a-d. The stiffness of the repaired transverse acetabular fracture construct as measured by the motion of the piston of the materials testing machine and the force applied by the piston is summarized in Figure 4a. We found that constructs with two column fixation were statistically stiffer than an anterior column plate alone. Only the posterior column plate with an anterior lag screw was statistically stiffer than the anterior locking plate. There was no statistical difference between the anterior locking plate and the anterior column plate with posterior lag screw. A construct of an anterior column plate with a posterior column lag screw (ACPLS) is 41% stiffer than the anterior column plate (ACP) alone (p = 0.0365) and 21% stiffer than the anterior column locking plate (LOCK) (p = 0.2485). A posterior column plate and an anterior column lag screw (PCPLS) is 53% stiffer than a single anterior column plate (ACP) (p = 0.0005) and 31% stiffer than an anterior column locking plate (LOCK) (p = 0.0008). There was no statistical difference between the single column fixation schemes (anterior column reconstruction plate with bicortical screws (ACP) and the anterior column reconstruction locking plate with unicortical screws (LOCK)(p = 0.248)), and there was also no statistical difference between the two column fixation schemes (anterior column plate/post column lag screw (ACPLS) and posterior column plate/anterior column lag screw (PCPLS)).

### Analysis of fracture displacement

Displacements at the fracture site reflect the stiffness of fixation that was measured by the displacement of the loading piston (Figure 4b). There was no statistically significant difference in fracture displacements between the single column fixation constructs, and there were no statistically significant differences in fracture displacements between the two column fixation constructs. The two column fixation constructs allowed about half the fracture displacement as single column fixation constructs. A graphical representation of the overall fracture movement (Figure 4c) reveals that most of the displacement measured for all fixation schemes was in the shear direction.

## Discussion

As new methods of biomechanical fixation of transverse acetabular fractures are introduced, studies are needed to compare their biomechanical strength with constructs that are well established. Our study was designed to compare the fixation stiffness of transverse acetabular fractures using anterior column locking plates, conventional anterior column plates, and plate-lag screw combinations.

For this study we chose polyurethane foam as an alternative test medium for human cancellous bone. These polyurethane foam pelvi are not intended to replicate the mechanical properties of human bone, however, they do provide consistent and uniform material with properties in the range of human cancellous bone. Polyurethane models allowed us to test a large number of pelvi that were required to complete this study with adequate power. Though the actual values of displacement and force that we report in this study may not represent the values that one would find in testing a bony pelvis, we believe the general findings of our study are applicable to the clinical situation.

The direction in which we chose to load our specimen matched the loading direction of previous studies, and thus allowed for the comparison of data [1,9,10]. This loading direction, however, represents one of an infinite number of possible loading directions for the hip. Rising from a chair, descending stairs, and other common clinical scenarios were not modeled in our study.

Our study revealed that two-column fixation constructs are significantly stiffer than a single column fixation construct with a conventional plate. We were not able to detect a significant difference between an anterior column locking plate (LOCK) and an anterior plate with posterior column lag screw (ACPLS). There was, however, a trend towards the ACPLS being stiffer than the LOCK. Only the posterior plate with anterior column lag screw (PCPLS) was significantly stiffer than the single column constructs.

In cases where a single column fixation may suffice, an anterior locking plate offers 16% more stiffness than a conventional plate. In addition, the locking construct provides some important advantages. First, locking plates do not depend on plate-bone contact and friction to achieve stability. Fracture fixation with a conventional plate relies on the compressive force provided by the screw head to the plate and the friction coefficient between plate and bone [3]. Insufficient compressive force from the screw head to the plate or insufficient friction between the plate and the bone will result in compromise of stability across the fracture site, and potential failure of fixation. The complex shape of the pelvis and the difficulty of the approach make achieving good plate-bone contact more difficult than when plating a long bone fracture. Plate contouring is not an issue when a locked plate is used as it achieves fixation as a fixed angle device and does not depend on plate-bone contact. Second, since similar fixation can be achieved with the locking plate using unicortical screws as with a conventional plate using bicortical screws, one would expect that the likelihood of iatrogenic neurovascular injury and joint penetration during pelvic and acetabular surgery would be reduced with placement of unicortical screws.

In conclusion, two column fixation provides the biomechanically stiffest construct for stabilization of transverse acetabular fractures, a finding that is consistent with previously published reports. We were not able to detect a statistical difference between a single anterior locking plate and an anterior plate with a posterior column lag screw. However, a posterior plate with anterior column lag screw was significantly stiffer than an anterior locking plate. We found a trend towards greater stiffness of the anterior locking plate compared to the conventional plate, but statistical significance was not reached.

## Conflict of interests

The authors declare that they have no competing interests.

## Authors' contributions

KK, ATL and DPL carried out the experiments, PL, MJB and NJG participated in the study's design and coordination and drafted the manuscript. All authors read and approved the final manuscript.
